# Intramedullary Conus Medullaris Tuberculoma: A Case Report and Review of the Literature

**DOI:** 10.3390/idr13010010

**Published:** 2021-01-15

**Authors:** Verajit Chotmongkol, Chinadol Wanitpongpun, Warinthorn Phuttharak, Sittichai Khamsai

**Affiliations:** 1 Department of Medicine, Faculty of Medicine, Khon Kaen University, Khon Kaen 40002, Thailand; vercho@kku.ac.th (V.C.); chinwa@kku.ac.th (C.W.); 2 Department of Radiology, Faculty of Medicine, Khon Kaen University, Khon Kaen 40002, Thailand; pwarin@kku.ac.th

**Keywords:** intramedullary tuberculoma, conus medullaris, adenosine deaminase, Froin’s syndrome

## Abstract

Intramedullary tuberculoma (IMT) of the conus medullaris is extremely rare. We present a case of intramedullary conus medullaris tuberculoma in which the diagnosis was based on there being very high levels of adenosine deaminase (ADA) in the patient’s cerebrospinal fluid (CSF) and improvement with antituberculous therapy. A 78-year-old man presented after having had a dull ache in both thighs and progressive paraparesis. The patient’s medical history included diffuse large B-cell lymphoma, which had undergone remission due to chemotherapy two years earlier, and long-term, well-controlled diabetes. A chest X-ray showed no evidence of tuberculosis. The results of CSF analysis were compatible with Froin’s syndrome. An initial diagnosis was made of an intramedullary tumor of the conus medullaris, based on magnetic resonance imaging (MRI). A myelotomy and multiple punch out biopsy were performed, and histopathology of the tissues revealed mild reactive gliosis. Due to the patient having high levels of CSF-ADA, IMT of the conus medullaris was suspected. The patient was treated with an 18-month course of antituberculous therapy. The dull ache gradually disappeared, and motor power improved slightly. A follow-up MRI of the lumbosacral (LS) spine revealed that the lesion had completely disappeared. Intramedullary tuberculoma of the conus medullaris should be considered in patients with underlying malignancy and no symptoms of systemic tuberculosis. CSF adenosine deaminase levels can be helpful in determining the presence of central nervous system tuberculosis when other systemic signs of disease are lacking.

## 1. Introduction

The most common type of neurotuberculosis is tuberculous meningitis (TBM). Early diagnosis and treatment with chemotherapy and active management of the complications are of great importance to prevent the irreversible neurologic sequel and death. A definite diagnosis of TBM depends on identifying *Mycobacterium tuberculosis* in the cerebrospinal fluid (CSF) by direct staining or culture. However, the diagnostic yield of CSF smears and cultures has been very low, and mycological cultures may take up to six weeks to yield results [1]. Spinal intramedullary tuberculoma (IMT) is an uncommon disease, with roughly 170 cases having been reported to date. The most common site of involvement is the thoracic cord. The condition occurs in relatively young patients and is often associated with extraspinal tuberculosis disease [2,3]. Intramedullary conus medullaris tuberculoma is extremely rare. Here, we report a case of IMT of the conus medullaris in an elderly man with underlying lymphoma and diabetes. A diagnosis was made based on there being a very high level of adenosine deaminase (ADA) in the patient’s cerebrospinal fluid (CSF) and improvement with antituberculous therapy.

## 2. Case Report

A 78-year-old man presented after having had a dull ache in both thighs for six weeks, and progressive paraparesis for one week. He had a history of diffuse large B-cell lymphoma with symptoms of generalized lymphadenopathy and splenomegaly, for which he had undergone a complete course of chemotherapy (R-CHOP), leading to disease remission two years prior. He also had long term, well-controlled diabetes. General and systemic examinations were normal. Motor power of both lower limbs was 1/5 with an absence of deep tendon reflexes and joint position sense. The remaining of the neurological examination was normal.

**Figure 1:**
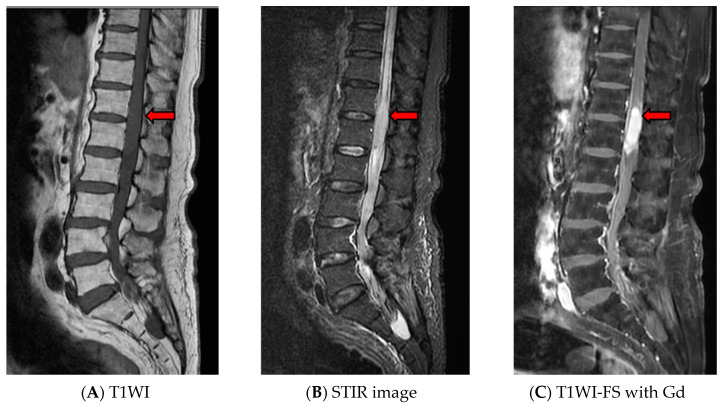
Magnetic resonance imaging of the lumbosacral spine revealed a 4.5 × 1.5 cm intramedullary expanding lesion at the T12-L1 level. The lesion exhibited isosignal intensity on the T1-weighted image (**A**) and hypersignal intensity on the Short tau inversion recovery (STIR) image (**B**) with homogeneous enhancement (**C**). Hypersignal intensity of the spinal cord above the lesion was also detected on the STIR image (**B**), which represented diffuse spinal cord edema.

Lumbosacral plexopathy was initially diagnosed, caused by infection or lymphoma involvement. A lumbar puncture was performed and xanthochromic CSF was found. Cerebrospinal fluid analysis revealed a white blood cell count of 2 cells/mm^3^, a protein concentration of 2181 mg/dl, and a glucose concentration of 57.9 mg/dl (concurrent a blood glucose concentration of 115 mg/dl). Gram stain, Ziehl–Neelsen stain, Indian ink preparation, cryptococcal antigen, and culture were all negative. The CSF culture, which was subsequently reported, was also negative for *M. tuberculosis*. Adenosine deaminase levels in the CSF will help in determining the presence of central nervous system tuberculosis, which was determined by an automated method, were 30.9 U/L. Cytopathological and flow cytometric analysis of the CSF demonstrated no evidence of malignant lymphoma. Due to the patient’s high CSF protein concentration but normal cell count (Froin’s syndrome), magnetic resonance imaging (MRI) of the lumbo-sacral (LS) spine was performed to rule out a spinal cord compression, and it showed a 4.5 × 1.5 cm intramedullary expanding lesion at the T12-L1 level. The lesion was isointense on T1-weighted and hyperintense on T2-weighted and short tau inversion recovery (STIR) images, and exhibited enhanced homogeneously after contrast administration. Diffuse spinal cord edema above the lesion was also detected (Figure 1). The results of a chest X-ray were within the normal limits. Anti-HIV was non-reactive. A T12-L1 laminectomy was performed, revealing an enlarged conus with an irregular surface. The nerve roots appeared normal. A right-side myelotomy and multiple punch out biopsy were performed. Histopathology of the tissues revealed mild reactive gliosis. Neither granuloma nor a tumor was found then Ziehl–Neelsen stain and tissue culture for *M.tuberculosis* were not performed. After the operation, the patient suffered from urinary retention, which did not resolve.

**Figure 2:**
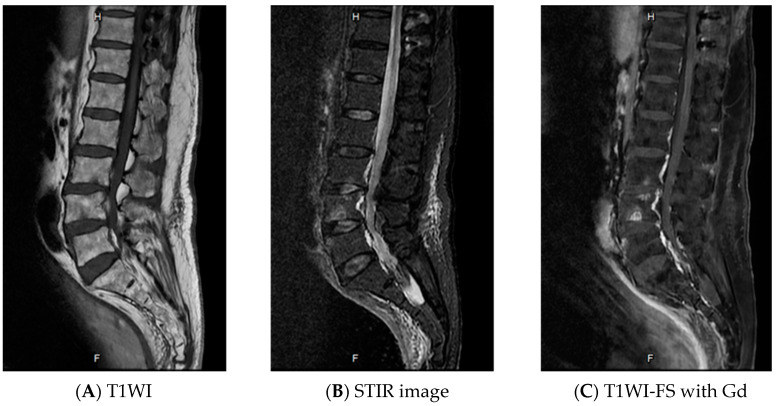
Magnetic resonance imaging of the lumbosacral spine at the 16th month of treatment showing complete resolution of the lesion.

Tuberculoma of the conus medullaris was suspected due to the patient having very high CSF-ADA levels [4]. Since Thailand is an endemic area of tuberculosis, we do not routinely use tuberculin skin test or QuantiFERON-TB Gold test for diagnosis of active tuberculous infection. The patient was treated with a combination of antituberculous drugs (isoniazid (I), rifampin (R), pyrazinamide (Z), ethambutol (E)), for two months, followed by IR for eight months, and a six-week course of steroids. The dull ache gradually disappeared, and motor power improved slightly. A follow-up MRI of the LS spine at the 10th month of treatment revealed a marked decreased in the size of the lesion to 0.37 × 0.53 cm. Treatment with IR drugs was administered for eight more months. The total duration of treatment was, thus, 18 months. A follow-up MRI of the LS spine revealed that the lesion had completely disappeared (Figure 2). At two years follow-up after completion of antituberculous therapy, the motor power of the left leg improved to grade 2–3/5, and the patient has been on permanent suprapubic cystostomy.

## 3. Discussion

Our patient was less likely to have central nervous system (CNS) relapse of a systemic lymphoma. A review of diffuse large B-cell lymphoma patients with CNS relapse by Villa et al. revealed that the use of R-CHOP appears to reduce the overall risk of CNS relapse, particularly in patients who achieve a complete response. The authors also showed that relapse in the CNS is a serious and most often fatal event. The median survival following CNS relapse (with treatment of CNS relapse) in patients who received the primary therapy with R-CHOP was 3.6 months [5].

Spinal tuberculosis commonly presents as tuberculous spondylitis and arachnoiditis [3]. Isolated without the involvement of overlying bone and meninges is rare, and the most common site of involvement is the thoracic part. Intramedullary tuberculoma of the conus medullaris is extremely rare, and to our knowledge, only about 14 cases have been reported to date in the English literature [6,7,8,9,10,11,12,13,14,15,16,17,18,19]. Table 1 summarized the clinical manifestations and outcomes of these cases, including the present case. Among the 15 cases, there were 12 in which the patients were male (80.0%). Patients’ ages range from 12 to 78 years (mean; 31.9 years), and the duration of symptoms varied from two days to six months. All of the patients had motor weakness of the lower limbs (one or both legs) and most of them had sensory impairment. Other manifestations were urinary symptoms, impotence, and back pain. These clinical presentations are not distinct from those of any other intramedullary mass lesion of the conus medullaris [20]. Associated tuberculosis elsewhere in the body was recorded in eight patients (53.3%). One patient had a history of pulmonary tuberculosis and had undergone antituberculous therapy. One patient developed spinal cord symptoms during treatment of tuberculous meningitis and intracranial tuberculoma, and one patient had had a tuberculous cerebellar abscess 10-months previously. Another two of the patients were HIV positive, and one had a history of lymphoma, which had undergone remission after a course of chemotherapy, and diabetes mellitus. The diagnoses were based on histopathology in eight patients, response to chemotherapy in five patients, lymphocytic CSF with high protein and low glucose level in one patient, and high levels of CSF-ADA in one patient. Surgical excision was performed in six patients (40.0%). The total duration of antituberculous therapy varied from six to 18 months. Most of the patients exhibited marked improvement after treatment.

Adenosine deaminase (ADA) is an enzyme involved in purine catabolism. It is considered as an indicator of cell-mediated immunity and is found mainly in T lymphocytes [21]. ADA level is also increased in nontuberculous meningitis, but it is marked significantly increased in tuberculous meningitis. So, detection of CSF-ADA activity in the diagnosis of TBM had a relatively high accuracy [22].

In cases such as these, MRI is the optimal method of investigation, as it is both sensitive and noninvasive. The MRI features of IMT can vary depending on the stage of tuberculoma formation (noncaseating, caseating with a solid center, and caseating with a liquid center). In its earliest stage (noncaseating), tuberculoma is characterized by severe inflammatory reaction with poor formation of the collageneous capsule. T1-weighted images show isosignal or slightly hyposignal intensity, and T2-weighted images show hypersignal intensity. The granuloma exhibits homogeneous enhancement after contrast administration on T1-weighted images. Later during the caseating stage, collagen becomes richer in the surrounding capsule, and rim enhancement becomes visible on an MRI after contrast administration. A solid caseating tuberculoma appears iso- to hypointense on T1-weighted images and hypointense center with an iso- to hyperintense rim on T2-weighted images. When the solid center of the caseating tuberculoma liquefies, T1-weighted images show hyposignal intensity and T2-weighted images show hypersignal intensity at the center with a hypointense rim [23] However, these findings are not specific to tuberculoma. The differential diagnoses for space-occupying lesions of intramedullary conus medullaris can be based on the presence of spinal intramedullary tumors (astrocytoma, ependymoma, metastasis, lymphoma) and granulomatous inflammations.

A diagnosis of tuberculous infection of the CNS can be based on AFB staining and CSF culture, subacute to chronic lymphocytic CSF with high protein and low glucose levels, histopathological findings, DNA genomic amplification using the polymerase chain reaction (PCR) method, high levels of CSF-ADA, the presence of extraneural tuberculosis foci elsewhere in the body, and responsiveness to antituberculous therapy [4,24,25]. In our case, the initial diagnosis was based on extremely high levels of ADA in the patient’s CSF.

There is, as of yet, no consensus with regard to the medical and surgical protocols to be employed in the management of spinal IMT. In practice, most researchers recommend antituberculous therapy, with or without corticosteroid, as the initial treatment when IMT is suspected. A combination of four drugs (isoniazid, rifampicin, pyrazinamide, and ethambutol or streptomycin) are administered over a period of two to three months followed by a combination of isoniazid and rifampicin. The total duration of treatment extends from 12 to 18 months. Patients with IMT often recover fully with non-surgical medical treatment alone. Surgery is reserved for cases in which: (1) the diagnosis is uncertain, (2) there is poor response to medical management, (3) there is progressive deterioration of neurological deficits during medical treatment, or (4) enlargement of the lesions with mass effect is observed on a follow-up MRI [26,27].

## 4. Conclusions

This is a report of a rare case of intramedullary conus medullaris tuberculoma in an elderly adult with underlying lymphoma and diabetes in which there was an absence of any evidence of systemic TB. CSF adenosine deaminase levels can be helpful in determining the presence of central nervous system tuberculosis when other systemic signs of disease are lacking.

## Figures and Tables

**Table 1:** Clinical features of patients with intramedullary tuberculoma of the conus medullaris.

Author	Age/Sex	Underlying Disease	Clinical Presentation and Duration of Symptoms	Associated TB	Diagnostic Method	Surgical Excision	Duration of Antituberculous Treatment	Clinical Outcome
Bradbury et al. (1980) [6]	26/M	No	Rt. Leg weakness, decreased sensation in the L5-S1 distribution duration: N/A	Pulmonary TB	Response to chemotherapy	No	N/A	Complete recovery
Choksey et al. (1989) [7]	31/F	No	Paraparesis, decreased sensation in the sacral dermatome, ascending to the L1-L2 dermatomes, urgency of micturition duration: 4 weeks	No	Histopathology	No	N/A	Marked improvement
Sie et al. (1994) [8]	24/M	No	Paraparesis, hypoesthesia in the sacral dermatomes, urinary urgency, impotence duration: N/A	No	Histopathology	No	N/A	Marked improvement
Dehoux et al. (1996) [9]	58/M	No	Fever, headache, confusion for 1 month, followed by back pain and legs, left hemiparesis, weakness of the right legs for 2 weeks	Tuberculous meningitis and cerebral tuberculoma	Lymphocytic CSF with high protein and low glucose level	No	N/A	Marked improvement
Suzer et al. (1998) [10]	20/M	No	Low back pain, urinary urgency, impotence, distal weakness of both legs, hypoesthesia in the sacral dermatome, ascending to L4 dermatome Duration: 10 days	No	Histopathology	Yes	N/A	Marked improvement
Parmar et al. (2000) [11]	35/M	HIV	Paraparesis Duration: N/A	Pulmonary and left ankle TB	Response to chemotherapy	No	18 months	improvement
Kemaloglu et al. (2001) [12]	32/M	No	Paraparesis and pain, urinary incontinence Duration: N/A	PulmonaryTB	Histopathology	Yes	12 months	Marked improvement
Tureyen (2002) [13]	46/M	No	Lt. leg weakness Duration: 7 days	History of PulmonaryTB	Histopathology	Yes	6 months	Complete recovery
Jaiswal et al. (2006) [14]	12/F	No	Low back pain, paraparesis with numbness, urinary incontinence Duration: 6 weeks	No	Histopathology	Yes	N/A	Marked improvement
Skoglund et al. (2007) [15]	21/M	No	Lumbar pain, sensory disturbance of both legs and perianal, lt. leg weakness, pain during miction, fever Duration: 2 months	PulmonaryTB	Response to chemotherapy	No	9 months	Marked improvement
Maamar et al. (2007) [16]	22/M	No	Paraparesis with paresthesia, urine retention, fever Duration: 6 months	No	Histopathology	Yes	12 months	Marked improvement
Lawler et al. (2013) [17]	12/F	HIV	Paraparesis with hyperaesthesia, urinary retention, febrile Duration: 2 days	Cerebellar abscess TB	Response to chemotherapy	No	N/A	Marked improvement
Sharoff et al. (2017) [18]	46/M	No	Low back pain, bladder incontinence, impotence, paraparesis and hypoesthesia below L3 Duration: 1 month	No	Histopathology	Yes	N/A	Complete recovery
Jaiswal et al. (2017) [19]	16/M	No	Back pain, paraparesis, impaired sensation below L1, fever Duration: 2 weeks	Pulmonary TB	Response to chemotherapy	No	N/A	Marked improvement
Present case	78/M	Lymphoma, diabetes mellitus	Paraparesis with dull ache Duration: 6 weeks	No	High level of CSF-ADA	No	18 months	Minimal improvement

TB: Tuberculosis, CSF: Cerebrospinal fluid, ADA: Adenosine deaminase, N/A: Not available.

## Abbreviations

IMT: intramedullary tuberculoma; ADA: adenosine deaminase; CSF: cerebrospinal fluid; MRI: magnetic resonance imaging; STIR: short tau inversion recovery; CNS: central nervous system.
